# Consumption of Deep-Fried Food and Its Association with Cardiovascular Risk Factors among First-Year Students in a Chilean University

**DOI:** 10.1155/2021/5591662

**Published:** 2021-05-07

**Authors:** Marcos Flores, Lucía Meyer, Pablo Jorquera, Patricio Castro, Carolina Saravia, Claudia Galdames, Sandra Orellana

**Affiliations:** ^1^Departamento de Ciencias Básicas, Facultad de Ciencias, Universidad Santo Tomás, Santiago, Chile; ^2^Escuela de Nutrición y Dietética, Facultad de Salud, Universidad Santo Tomás, Santiago, Chile; ^3^Escuela de Kinesiología, Facultad de Salud, Universidad Santo Tomás, Santiago, Chile; ^4^Hospital Regional de Talca, Talca, Chile

## Abstract

The increase in the consumption of deep-fried foods (DFFs) from street vendors in Chile affects body weight and health. However, the actual consumption frequency of these types of foods by population and their relationship with cardiovascular risk factors (CVRFs) is unknown. The consumption frequency of eight DFFs sold in street vendors was obtained through a questionnaire. CVRFs were determined through survey and anthropometric measurements. The prevalence of high values of CVRF was determined regarding gender and frequency of consumption of DFF monthly. 66% of the population consumed DFF more than 4 times monthly; each individual would consume about 4 (3.83) servings/week and with 81% of preference of products sold from street vendors. 89.2% of the population exhibited a family history of CVD. From the means comparison application, although the values obtained from CVRF were mostly within normal ranges at the population level, there were significant differences in CVRF according to gender in body mass index, waist circumference, systolic pressure, and sedentary. The prevalence of elevated values of CVRF according to gender was higher in men than in women in 7 of 12 of the parameters of CVF. The prevalence of elevated values of CVRF according to the frequency of consumption of DFF monthly was higher when students consume DFF more than 4 times monthly. The 12.7% of the population had between 3 or 4 CVRFs and consumed high quantities of DFF, preferably sold by street vendors near the university. This situation is worrying since the caloric and nutritional content is low and could generate a tendency to acquire cardiovascular pathologies in the medium term.

## 1. Introduction

According to the National Health Survey (NHS) 2009-2010, it appears that the Chilean population between 15 and 64 years of age presented 64.5% overweight or obesity, 88.6% sedentary lifestyle, and 35% metabolic syndrome (MS). This reflects that the profile of obesity and chronic noncommunicable diseases in Chile follows the trend of a developing country [1, 2], with a marked increase in the body mass index (BMI), both for men and women in the last decades, being higher in women [3].

In the last decades, the Chilean population has deeply modified their behaviors and alimentary habits. This situation applies to the group of university students, who mostly live far from their family homes and do not dedicate time to healthy cooking and exercise. This results in an increase of sedentary lifestyles and in the replacement of healthy foods by food products sold in street vendors and established commerce. These food products have high caloric density, low nutritional values, and high flavor concentrations and satisfy the appetite in a short-term way. This situation is directly related to some studies that show student populations that have increased their body mass between one and two kilograms per year after entering university, even up to ten kilograms during their stay in the university [3–6]. In this way, this community would follow the trend of high consumption of fast food, spending during 2016 about 21 billion Chilean pesos of the 157 billion of the budget designated by the Chilean State to the FGUE grant (Food Grant for University Education) according to data provided by the Transparency Law. This information is worrying if it is considered that this scholarship is granted to university students of limited incomes, in order to consume healthy food and not “junk or fast food.” According to the Food Labeling Law 20.060 of Chile, junk or fast food's contents of energy, saturated fat, sugar, and sodium are off the healthy limits [7].

The “junk” or “fast” food consumed by this population mainly includes products subjected to deep-frying processes, whose sale has increased in recent years both in the established trade and in street vendors. The latter considers selling food in vehicles or small sale stands located around universities and colleges, poorly controlled or supervised, often without access to potable water, with low technical and sanitary considerations, unregulated nutrition labeling, and a complex control by the health authority [8–10].

The Obesity Observatory (“Observacción”), a new Chilean organization dedicated to the study of obesity and its implications in the population, has found that the consumption of diets rich in saturated fats and trans unsaturated fats [11] negatively modifies the human lipid profile by increasing the risk of developing diabetes, overweight, obesity, and cardiovascular diseases [12–16]. In addition, specifically in Chile, cardiovascular diseases are the main cause of death in that population. The 2011–2020 goals proposed by the Chilean Ministry of Health and its associated programs consider reducing cardiovascular risk factors (CVRF) by promoting the improvement of eating habits and physical activity, which is essential to generate a healthy lifestyle [17]. At the same time, these guidelines should be complemented with a reduction of fried foods and fatty foods consumption, since they are considered food products that can modify the lipid profile if they are highly consumed, which would constitute a potential cardiovascular risk factor affecting negatively the health of the population [18–20].

Studies in Chile have analyzed the lifestyle, nutritional status, and risk behaviors of school students [21, 22] and university students [5, 23–25], highlighting the latter. The university population is a highly vulnerable group in order to develop risk behaviors and habits, coupled with scientific researches that show that their diet is inappropriate with a high fat intake and low dietary fiber intake. These backgrounds would increase the trend to develop some pathologies such as overweight, obesity, and cardiovascular diseases (CVDs). This situation increases the cost of health services and decreases productivity, a situation similar to that found in other countries such as Argentina, Mexico, and Spain [26–28], with also the possibility of deriving through time in metabolic syndrome (MS) due to the coexistence of several risk factors [29, 30]. Therefore, university time is a very important period to educate and help develop healthy eating habits that will be extremely important in the future health of each person.

Although, in Chile, it has been determined that 39.3% of the national population is overweight and 25.1% is obese, these parameters are not clear in the population of university students who, for example, are entering the first year of university. Furthermore, there is no much information about the quality of fats present in deep-fried foods (DFFs) that this population consumes and their consumption frequency, especially foods sold by street vendors such as French fries or chips and different products derived from fried doughs with and without filling. In this line and considering these factors, the central aim of this study was to analyze the presence of CVRF in first-year students at the Universidad Santo Tomás, Talca campus, who are faced with a change in their lifestyles, due to situations of self-preparation of food, consumption of fast food, and access to DFF purchased from street vendors.

## 2. Materials and Methods

The present investigation, carried out during the second half of 2014, is a quantitative, descriptive, and cross-sectional study, focused on the presence of CVRF in a representative sample of students from the Universidad Santo Tomás campus Talca who consume DFF from street vendors. This study was validated and approved by professionals in food and health areas, nutrition and sciences in general, both internal and external to the institution. In addition, the Ethical Investigation Committee of the Universidad Santo Tomás accepted this study with code CE N°77/2013, during December 2013.

*Data Collection*. The sample size calculation for this study considers the type of sample, parameter to be estimated, admissible sampling error, population variance, and confidence level. As the size of the population was known, the sample size was determined by the following formula [31, 32] with a confidence level of 90% and a 5% error:

(1) $$\begin{array}{c} n=\frac{\left(Z_{\alpha }^{2}*PQN\right)}{e^{2}\left(N-1\right)+Z_{\alpha }^{2}*PQ}, \end{array}$$

where Z*α* corresponds to the *Z* value (of the chosen confidence level of 90%) = 1.64, *P* is the proportion of a category of the variable (0.5), and *Q* is the proportion that does not present the study characteristic (1 − *P* = 0.5). And since the expected proportion is unknown, it is decided to use the conservation criterion where *P* = *Q* = 0.5 = 50%. *N* is the size of the universe of the total population = 1000, and *E* is the maximum sample error (equivalent to 5%) = 0.05.

Therefore, replacing the previous values in the sample size formula for this study, the following was obtained:

(2) $$\begin{array}{c} n=\frac{{\left(1.64\right)}^{2}\times 0.5\times 0.5\times 100}{{\left(0.5\right)}^{2}\times 999+\left(1.64\right)2\times 0.5\times 0.5}=212. \end{array}$$

The study sample for this research considered 212 of the 1002 students (32 men and 180 women) belonging to the 2014 admission process and first year of university, obtained through a nonprobabilistic sampling using snowball, where an invitation was made to all the target students through the different school directors, as well as invitations to participate in each course directly to the students by the researchers in charge of the research, where certain days of the week were summoned to go to the science laboratory to participate in the study. After the students were assessed, each student was asked to have access to other students in order to achieve the actual sample size. This type of sampling was selected due to the complexity of the access to first-year university students, where many of them do not know this type of studies [32, 33]. The students voluntarily arrived at the laboratory where they signed a letter of consent to continue the process of collecting personal information.

In agreement with previous background, the following inclusion criteria were determined for those students who were considered within the sample and who had to comply with signed informed consent and belonging to the first year of university. In the present study, those who presented one of the following exclusion criteria were not considered in the sample:

- Students who left the university during the course of the investigation
- Students who presented pathologies that limited the taking of blood samples (allergies, tegumentary hypersensitivity, excessive bleeding, among others)
- Pregnant students due to the alterations that are caused in anthropometric measurements and biochemical profile

Personal and family history, socioeconomic and sociocultural data, morbidity history, physical activity levels, blood pressure, blood glucose, lipid profile, and anthropometric measurements (weight, height, and waist circumference) among others were collected in an initial form that was validated by biology, social sciences, and nutrition experts who evaluated the ability of the instrument to collect and evaluate all the dimensions that were intended to be measured in the present study.

In order to evaluate the physical activity levels [34], a questionnaire from the Second National Survey on Quality of Life and Health (ENCAVI 2006) was applied. It considered the student as sedentary if he or she did not practice any sport or physical activity during the last month and as an active student if he or she performed 30 minutes of physical exercises at least 3 times a week. The anthropometric measures, such as weight and height [23, 35], were determined using a scale with a mechanical height meter for adults, calibrated by SECA ®, graduated in kilograms (kg) and grams (g) to measure weight and in meters (m) and centimeters (cm) to measure height. From these data, the general nutritional status [23, 35] of each student was evaluated using the Quetelet Index or Body Mass Index (BMI), with a normal BMI: range 18.5–24.9 kg/m^2^; overweight: range 25–29.9 kg/m^2^; and obesity: ≥30 kg/m^2^. The waist circumference (WC) was determined with a SECA® branded self-retracting tape measure in centimeter (cm), considering the point between the anterosuperior iliac spine and the lower costal margin as a midpoint. Men were considered obese if their WC was higher than 88 cm, and for women, WC higher than 83 cm was considered obese [23].

Blood pressure [35] was measured according to the clinical guidelines for hypertension of Chile (MINSAL), recording systolic blood pressure/diastolic blood pressure and considering the normal state: <130/<80 mmHg, prehypertensive: 130–140/80–90 mmHg, and hypertensive: >140/90 mmHg. Fasting blood glucose level was measured with a CareSens brand equipment (Refined CareSens N Meter) which considered normal values: 70–100 mg/dl and altered values: >100 mg/dl, according to the equipment instructions.

The lipid profile (total cholesterol, HDL cholesterol, LDL cholesterol, and triglycerides) was evaluated through blood samples taken in a clinical laboratory using a colorimetric enzymatic method in Cobas c311 equipment and its Roche brand reagents; this test was taken in a fasting condition of 12 hours. This methodology was subjected daily to quality control using BioRad reagents. Normal values were considered for total cholesterol <200 mg/dl, HDL cholesterol >40 mg/dl, LDL cholesterol <100 mg/dl, and triglycerides <150 mg/dl.

The metabolic syndrome [23] was determined by the presence of at least three CVRF in each student. The considered parameters were as follows: increase body mass index indicating overweight or obesity (≥25 kg/m^2^), increase waist circumference indicating abdominal obesity (in men > 88 cm and in women > 83 cm), fasting blood glucose level (>100 mg/dl), increased blood pressure or hypertension (>140/90 mmHg), decreased HDL cholesterol (<40 mg/dl), increased LDL cholesterol (>100 mg/dl), increased total cholesterol (>200 mg/dl), hypertriglyceridemia (>150 mg/dl), and sedentary and moderate and high consumption of DFF.

Finally, eating habits, food preferences, and related behaviors were obtained by applying the Quantitative Consumption Trend, validated by experts in food, nutrition, and health areas, in the same way as for the initial form. This questionnaire was proposed based on the internal research project of the Universidad Santo Tomás and validated by the institutional Ethics Committee. The consumption frequency of DFF was determined (French fries, fried empanadas (wheat flour dough filled with cheese or meat and onion), spring rolls (roll of Philo rice flour dough filled with meat and cabbage), sopaipillas (wheat flour dough with pumpkin), calzones rotos (fried flour dough with baking powder, egg, and sugar flower), churros (wheat flour dough cooked in oil), donuts (sweet wheat flour doughs), and berlines (sweet wheat flour dough stuffed with sweet cream)) in students' population sold in street vendors considering DFF type, number of servings and times consumed, and type-place commerce. The classification of consumption patterns was high consumption (>4 times a month), moderate consumption (3-4 times a month), low consumption (1-2 times a month), and no consumption (0 times a month).

*Statistical Analysis*. We determined whether the data from the different variables showed normal distribution through the Kolmogorov-Smirnov normality test when *n* ≥ 50 observations. For the comparison of the quantitative parametric variables between two groups, Student's *t*-test was used. In the case of nonparametric variables, the Mann–Whitney *U* test was used when comparing two variables. All analyses were performed with the SPSS program, version 19 for Windows with a *p* ≤ 0.05.

## 3. Results

**Figure 1:**
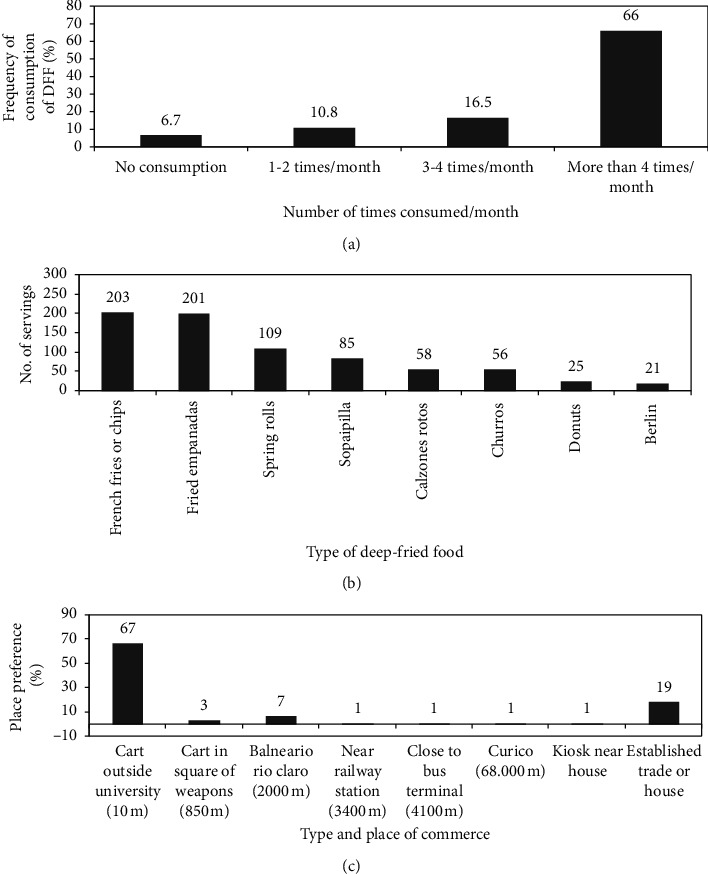
Analysis of the consumption of DFF expended in street commerce by the university students' population. (a) Frequency of consumption of DFF according to 4 categories: no consumption, 1-2 servings per month, 3-4 servings per month, and more than 4 times per month. (b) Total population preferred consumption of DFF expended in street commerce according to the number of servings/week. (c) Places (itinerant and established) of DFF sale that are preferred by this population and those prepared at home.

The sample of 212 university students (32 men and 180 women) who voluntarily participated, were evaluated in different CVRF and surveyed about their frequency of consumption of DFF sold in street vendors. Of the considered sample, 93.3% consumed these foods and 66% of the 212 students reported consuming them more than 4 times a month (Figure 1(a)). From the different types of DFF sold in street vendors, we determined which this group of students prefers. Then, we organized the number of servings consumed per week and sorted them in decreasing order: French fries, fried empanadas, spring rolls, sopaipillas, calzones rotos, churros, donuts, and berlines.

Of the sample of 212 students surveyed, with respect to their weekly DFF consumption statement, only 6.7% (14 students) did not consume this type of food and the remaining sample of 198 students consumed them in high quantities or servings (Figure 1(a)). They consumed collectively 203 servings of French fries (chips) and ∼201 servings of fried empanadas a week, followed by 109 servings of spring rolls, ∼85 servings of sopaipillas, 58 servings of calzones rotos, ∼56 servings of churros, ∼25 servings of donuts, and ∼21 servings of berlines (Figure 1(b)). Therefore, the 198 students consumed in total ∼758 servings of DFF per week (Figure 1(b)) which is quite high for this population, since each individual consumed an average of 4 (3.83) servings per week. At this point, it is essential to mention that the size of each DFF is regular, and its composition and nutritional value is a little different, even in the amount of oil absorbed in the frying process. In spite of this, the average number of servings of these DFFs consumed by each individual of the 198 students was determined.

We also determined the places of the established commerce and street vendors that sell DFF and that are preferred by this population. Of the 198 students who consume DFF, 19% generated these foods at home or were bought in the established commerce. The remaining 81% bought in the street vendors, preferentially in the university surroundings, since it saves time of transfer when being to 10 meters of the university (Figure 1(c)).

Of the 212 students surveyed (Table 1), only 6.1% presented morbidity of CVD (13 students), represented with one man (3.1% of male population) and 12 women (6.7% of female population). However, the highest prevalence corresponds to a family history of CVD with 89.2% (189 students), being slightly higher in men (90.6%, 29 of 32 students) than in women (88.9%, 160 of 180 students). These parameters should be considered as risk factors along with others (data not shown: 32.1% of the population was considered smoker) that may add up and lead to a possible cardiovascular pathology (Table 1).

Of the 212 students surveyed, only 32 were men (15.1%) and 180 were women (84.9%), showing a great disproportion between genders. This situation provided a real image of the representative sample of the population, because the students surveyed belonged mainly to careers in the health area where the students are mainly women. From the comparison between CVRF and the gender variable (Table 2), BMI values of 26.2 kg/m^2^ for men and 24.2 kg/m^2^ for women, WC of 86.6 cm for men and 76.7 cm for women, and systolic pressure of 122.4 mmHg for men and 114.7 mmHg for women were found to be significantly different being higher in men than in women (*p* ≤ 0.05). The sedentary with 5 men and 78 women is significantly different being higher in women than in men (*p* ≤ 0.05). The variables blood glucose, diastolic pressure, HDL cholesterol, LDL cholesterol, total cholesterol, triglycerides, number of students with MS, and number of students with moderate to high consumption of DFF did not show significant differences between genders (*p* ≥ 0.05, Table 2).

We determined the prevalence of high CVRF values according to gender (Table 3). The results consider the following: 82.5% of the population consumes moderately to highly of DFF, 39.2% is sedentary, 34% presented elevated values of BMI, 33.5% presented high LDL cholesterol, and 22.6% of the population exhibited elevated waist circumference. It is important to mention that 12.7% of the students surveyed presented three or four altered CVRF (classified as the presence of metabolic syndrome) according to the criteria established in the clinical guidelines of the Ministry of Health of Chile. Seven of 12 high values CVFR were higher in men than in women (BMI, WC, Blood glycemia, SP, DP, HDL cholesterol, and LDL cholesterol). The 5 variables of total cholesterol, triglycerides, with MS, sedentary, and moderate to high consumption of DFF were higher in women than in men. Finally, when analyzing the relationship between the prevalence of elevated CVRF values and the consumption frequency of DFF monthly (Table 4), it was observed that the population calculated in percentage that had a moderate to high consumption of DFF is concentrated on the elevated values of CVRF.

## 4. Discussion

The fatty foods intake mainly associated with the “fast food” group by the world population has increased exponentially in the last decade and Chile is not the exception. According to the National Survey of Food Consumption (NSFC), 2010-2011, in Chile, there is excessive consumption of calories and saturated fats in all age groups [11]. Several studies support the direct relationship between the appearance of CVRF and fat consumption [5, 7, 17, 27, 30, 36].

Particularly, the population of university students represents an important pole of study because of their high susceptibility to being large consumers of fats [5]. In this context, the study was projected to deliver a real-time image of what is happening in students of the Universidad Santo Tomás campus Talca. It is verified that the analyzed population has a high consumption of DFF, consuming them more than four times monthly and preferably a lot of servings of French fries or chips and fried empanadas. It is worth mentioning that students preferentially acquire these products in the street vendors close to the university (10 m away), since it offers economic DFF, high caloric power, short distance, and quick or no preparation at all. Unfortunately, the consumption of DFF sustained over time could lead to serious health problems in this young population.

Although there are no scientific publications on the real consumption of the Chilean population of DFF, a study presented at the Universidad San Sebastian in Chile, as part of the talk “street food and other concerns that arise from the research in nutrition in Portugal” [37], explained that the sopaipillas are the fried foods more consumed in Chile for sale in street vendors, contrary to what happens in our study with a relative consumption.

Although the analyzed population is in its first year of university life, certain behaviors and/or risk factors were found that could become the cause of the appearance of a noncommunicable disease or even CVD in the near future, if the pattern keeps repeating itself in a constant way. This is reflected in the different CVRF analyzed in the total sample, constituting one of the highest prevalence in the family history of CVD (89.2%, Table 1), doubling the value of 41.8% presented by Chilean students of the Universidad Santo Tomás campus Temuco [23] and 44.6% according to Brazilian first-year students from Universities in São Paulo [38]. According to gender differences, it is slightly higher in men (90.6%) than in women (88.9%), unlike the values of 45.5% and 44.4% reported by Alarcón et al. [23] and of 50.9% and 37.5% by Costa et al. [38]. This parameter should be considered as a risk factor along with others that may add up and lead to a possible cardiovascular pathology.

From the general analysis of the mean values of parameters of CVRF evaluated in this population and with respect to gender (Table 2), it is clear that their results are within normal ranges, although the large number of students who consume moderate and high amounts of DFF, especially in women, is worrisome. Specifically, the results indicate that the BMI, WC, and systolic pressure were observed of significant differences between genders, presenting higher values for men than for women. BMI measurements (26.2 kg/m^2^ for men and 24.2 kg/m^2^) are a little higher compared to other studies evaluated in the Chilean (24.06 kg/m^2^ for men and 24.23 kg/m^2^ for women) and Brazilian student population (23.8 kg/m^2^ and 22.9 kg/m^2^). WC measurements (86.6 cm for men and 76.7 cm for women) were lower than another study in the Chilean student population (87.37 cm for men and 85.61 cm for Chilean women) and higher than in the Brazilian population (79.8 cm for men and 78.6 cm women) [23, 38]. Measurements of the systolic pressure (122.4 mmHg for men and 114.7 mmHg for women) are higher than those found in other Chilean university populations (109.47mHg for men and 104.2 mmHg for women) [23].

The prevalence of high values of CVRF according to gender, above the limits established in each case, was studied as indicators of risk to the cardiovascular system (Table 3). High values of certain parameters at the level of the analyzed population were determined such as BMI (34%), WC (22.6%), LDL cholesterol (33.5%), sedentary (39.2%), and moderate to high consumption of DFF (82.5%). The results indicate that 7 of the 12 analyzed parameters are higher in men than in women (BMI, WC, blood glucose level, systolic and diastolic pressure, and HDL and LDL cholesterol).

A possible cause of illness in the future is the combined prevalence of overweight and obesity, which can be determined by the BMI indicator. If the 34% observed in this population is compared with other results, it was found that they are higher in other Chilean students' population (28.6%) [23], Mexican students (30.7%) [26], Colombian students (23.6%) [39]. In contrast, a Chilean population of students of Universidad de la Frontera presented 35% overweight and obesity [40], 38.2% in Brazilian students [41], and 53.4% in university students in Ghana [42]. The fact that overweight and obesity increase with age and considering that the surveyed population corresponds to first-year students is a complex scenario with these values. Therefore, it would be essential to take preventive measures in the future to control both conditions. In addition to this, abdominal obesity is a good indicator of cardiovascular risk evaluated by WC; it is of special interest to worry about 22.6% of this population, since this parameter increases its value with age, so it is vital to analyze it in time. Regarding the lipid profile, this population exhibits a high value of prevalence of 33.5% LDL cholesterol, higher than 7.3% of other Chilean students [40], but lower than 43.9% of Chilean students [25] and 50.3% Colombian students.

39.2% of the population presented a sedentary lifestyle. In this study, this value is higher than that presented in students of the Universidad Autónoma de Mexico with 27.85% [26] and that studied by Costa et al. with 35.7% [38] but much lower compared to 91.5% of the population of the Universidad de Talca [25], 87.8% in students from the Universidad Austral de Valdivia [24], 70.6% of Universidad Santo Tomás campus Temuco [23], and 82.2% of Chilean population between 17 and 24 years according to the National Health Survey (2006) [35]. However, a similar trend of physical inactivity with significant differences was observed in all the studies, predominating more in women than men. This could be because men practice more physical activities, especially during the weekends, while women find it more difficult. It is for this reason that women with regard to this risk factor are more predisposed to develop a noncommunicable disease such as cardiovascular and/or metabolic, although this factor is modifiable when promoting physical activity. However, the 12.7% of the sample surveyed had 3 or 4 CVRF, relatively comparable to 9.6% of the Universidad Santo Tomás campus Temuco population [23] but highly over the 3.4% of Mexican students [26], which constitutes a conclusive antecedent of the significant prevalence of CVRF in the sample analyzed. Complementarily, 82.5% population consumes moderate and high DFF, being higher consumption in women (83.3%) than in men (78.1%) (Table 3).

The prevalence of high values of CVRF was analyzed according to the frequency of consumption of DFF monthly (Table 4). In all the parameters of CVRF analyzed, it is observed that a significant number of students who consume DFF do so more than 4 times a month, if compared with the rest of the consumption frequencies. This situation is serious if such tendency continues to repeat in time. It is considered that a greater consumption of fatty foods (saturated and trans fats) and in some cases subjected to frying in depth can produce over time an increase in the lipid profile, the risk of coronary heart disease, and even the appearance of some types of cancers [18].

Among the limitations of the study that could be considered is the disproportionality of the sample involving more women than men, whose explanation lies in the largest number of women enrolled in careers in the area of health during the first year in the institution. Another point would be the configuration of the sample voluntarily, not being able to control the motives of those students who did not want to participate in the study. Also consider the time needed to complete surveys (between 20 and 25 minutes) plus the time provided for evaluation of lipid profile and some anthropometric measurements, which made it difficult for the student to participate. And finally, as the study addressed the consumption of DFF mainly acquired in the street vendors and its association with CVRF, it was difficult to determine exactly the purchase of these foods in certain places, since many times as sellers do not have municipal permits, they move constantly, affecting the development of the study.

## 5. Conclusions

This research is one of the first published studies in Chile of CVRF in university students consuming foods subjected to deep frying or DFF. This mostly female population studied presented an important family history of CVD and certain relatively normal CVFR parameters (BMI, WC, blood glucose level, systolic and diastolic pressure, HDL and LDL cholesterol, total cholesterol, and triglycerides) with significant differences at the gender level in BMI, WC, and systolic pressure. From the study of the prevalence of high values of CVRF evaluated according to gender, 7 of 12 parameters analyzed were higher in men than women, standing out BMI, WC, and LDL cholesterol.

In the group of evaluated students with metabolic syndrome (MS, defined by the presence of 3 or 4 CVRF), sedentary lifestyle, and a moderate to high consumption of DFF, it was determined that the parameters were higher in the female population than in the male, being the most significant difference in a sedentary lifestyle.

And from the prevalence of high values of CVRF according to the frequency of consumption of DFF, it was determined that the population consumes more than 4 times a month DFF at the level of all the CVRF analyzed, which is surprising if this trend is maintained in time. This could possibly derive in addition to the family background to such students being able to increase their CVRF.

This study demonstrated that in a young population (between 18 and 29 years old) in its first year of university, 12.7% had metabolic syndrome associated with the presence of 3-4 CVRFs, whose value can change over time if they increase the values above the normal ranges of BMI, sedentary lifestyle, hypertension, lack of control in the lipid profile, and continuity with high consumption of DFF food. Starting from the base that most of the students surveyed had a family history of CVD, it may constitute a pole of young individuals with an increased cardiovascular risk in the medium term, especially if they continue to have risky dietary behaviors such as high consumption of fats purchased in street vendors and even established commerce. However, a large percentage of the evaluated population consumes significant amounts of DFF from street vendors at low cost and fast attention, without permanent sanitary control, but located in the vicinity of the university. If this consumption remains in time and the number of acquired servings increases, students will quickly increase the appearance of CVRF, which in turn can generate the presence in the future of noncommunicable and cardiovascular pathologies.

## Figures and Tables

**Table 1:** Morbidity history and family history of cardiovascular disease in the examined student population and in the male group and female group.

Parameter	Total = 212		Men = 32		Women = 180	
	*n*	%	*n*	%	*n*	%
Morbidity history of CVD	13	6.1	1	3.1	12	6.7
Family history of CVD	189	89.2	29	90.6	160	88.9

Data presented as *n* (number of students) and % (percentage). CVD: cardiovascular diseases.

**Table 2:** Cardiovascular risk factors (mean +standard deviation) in the male and female students examined, in addition to the percentage of students with MS, sedentary, and moderate and high consumption of DFF.

CVRF	Total *n* = 212	Men *n* = 32	Women *n* = 180	*p*
	Mean ± SD	Mean ± SD	Mean ± SD	
BMI (kg/m^2^)	24.5 ± 4.05	26.2 ± 4.6	24.2 ± 3.9	0.014 ^*∗*^
Waist circumference (cm)	78.0 ± 10.1	86.6 ± 11.0	76.7 ± 9.4	0.000 ^*∗*^
Glycemia (mg/dl)	92.5 ± 9.9	93.1 ± 7.7	92.4 ± 10.2	0.720
Systolic pressure (mmHg)	115.9 ± 9.9	122.4 ± 10.3	114.7 ± 9.4	0.000 ^*∗*^
Diastolic pressure (mmHg)	79.0 ± 8.2	78.6 ± 8.9	79.1 ± 8.0	0.750
HDL cholesterol (mg/dl)	53.9 ± 12.3	49.1 ± 11.2	55.5 ± 12.4	0.069
LDL cholesterol (mg/dl)	91.4 ± 29.7	90.8 ± 33.4	91.6 ± 28.7	0.926
Total cholesterol (mg/dl)	167.2 ± 31.6	160.9 ± 32.2	169.3 ± 31.5	0.359
Triglycerides (mg/dl)	115.8 ± 65.9	107.2 ± 26.6	118.6 ± 74.4	0.653
With MS (*n* students)	27	2 (6.3%)	25 (13.9%)	0.537
Sedentary (*n* students)	83	5 (15.6%)	78 (43.3%)	0.003 ^*∗*^
Moderate and high consumption of DFF (*n* students)	175	25 (78.1%)	150 (83.3%)	0.513

Data presented as mean ± standard deviation (SD). For the comparison of parametric variables between two groups, Student's *t*-test was used, and in the case of nonparametric variables, the Mann–Whitney *U* test was used.  ^*∗*^ There is statistical significance *p* < 0.05. n = number of students. CVRF: cardiovascular risk factors. BMI: body mass index. HDL cholesterol: high-density lipoprotein cholesterol. LDL cholesterol: low-density lipoprotein cholesterol. With MS: with metabolic syndrome. DFF: deep-fried foods.

**Table 3:** The prevalence of elevated values of CVRF according to gender.

CVRF	Total = 212		Men = 32		Women = 180	
	*n*	%	*n*	%	*n*	%
BMI (≥25 kg/m^2^)	72	34.0	15	46.9	57	31.7
Waist circumference	50	22.6	11	34.4	39	21.7
>88 cm (man)						
>83 cm (woman)						
Glycemia (>100 mg/dl)	32	15.1	5	15.6	27	15.0
Systolic pressure (>130 mmHg)	19	9.0	7	21.9	12	6.7
Diastolic pressure (>90 mmHg)	14	6.6	3	9.4	11	6.1
HDL cholesterol (<40 mg/dl)	23	10.8	13	40.6	10	5.6
LDL cholesterol (>100 mg/d/	71	33.5	16	50	55	30.6
Total cholesterol (>200 mg/dl)	29	13.7	3	9.4	26	14.4
Triglycerides (>150 mg/dl)	32	15.1	3	9.4	29	16.1
With MS	27	12.7	2	6.3	25	13.9
Sedentary	83	39.2	5	15.6	78	43.3
Moderate and high consumption of DFF	175	82.5	25	78.1	150	83.3

Data presented as *n* (number of students) and % (percentage). CVRF: cardiovascular risk factors. BMI: body mass index. HDL cholesterol: high-density lipoprotein cholesterol. LDL cholesterol: low-density lipoprotein cholesterol. With MS: with metabolic syndrome. DFF: deep-fried foods.

**Table 4:** The prevalence of elevated values of CVRF according to the frequency of consumption of fried foods monthly.

CVRF	High consumption (>4 times)		Moderate consumption (3-4 times)		Low consumption (1-2 times)		No consumption (0 times)	
	*n*	%	*n*	%	*n*	%	*n*	%
BMI (≥25 kg/m^2^)	44	61.1	11	15.3	12	16.7	5	6.9
Waist circumference	32	64.0	7	14.0	6	12.0	5	10.0
>88 cm (man)								
>83 cm (woman)								
Glycemia (>100 mg/dl)	23	71.9	4	12.5	4	12.5	1	3.1
Systolic pressure (>130 mmHg)	10	52.6	4	21.1	3	15.8	2	10.5
Diastolic pressure (>90 mmHg)	9	64.3	4	28.6	0	0.0	1	7.1
HDL cholesterol (<40 mg/dl)	11	47.9	6	26.1	3	13.0	3	13.0
LDL cholesterol (>100 mg/dl)	45	63.3	8	11.3	9	12.7	9	12.7
Total cholesterol >200 mg/dl	23	79.3	2	6.9	2	6.9	2	6.9
Triglycerides (>150150/dl)	28	87.4	2	6.3	0	0.0	2	6.3
With MS	15	55.6	8	29.6	2	7.4	2	7.4
Sedentary	56	67.5	15	18.1	10	12.0	2	2.4

Data presented as *n* (number of students) and % (percentage). CVRF: cardiovascular risk factors. HDL cholesterol: high-density lipoprotein cholesterol. LDL cholesterol: low-density lipoprotein cholesterol. With MS: with metabolic syndrome. DFF: deep-fried foods.

## Data Availability

The survey data used to support the findings of this study are available from the corresponding author upon request.
